# Effect of tuina on sleep quality, psychological state and neurotransmitter level in patients with insomnia: a systematic review and meta-analysis

**DOI:** 10.3389/fneur.2024.1273194

**Published:** 2024-02-21

**Authors:** Zhen Wang, Hui Xu, Zheng Wang, Hang Zhou, Lijuan Zhang, Yu Wang, Miaoxiu Li, Yunfeng Zhou

**Affiliations:** ^1^College of Acupuncture and Massage, Henan University of Chinese Medicine, Zhengzhou, China; ^2^Tuina Department, The Third Affiliated Hospital of Henan University of Chinese Medicine, Zhengzhou, China; ^3^Central Hospital of Jiaozuo, Jiaozuo, China; ^4^College of Computer Science, Xidian University, Xian, China; ^5^College of Acupuncture and Massage, Shanghai University of Chinese Medicine, Shanghai, China

**Keywords:** tuina, insomnia, sleep quality, anxiety-depressive state, neurotransmitter, meta-analysis

## Abstract

**Background:**

Abnormal psychological state and neurotransmitter levels are important factors affecting sleep quality. Numerous studies have shown that tuina can improve the symptoms of sleep disorders in patients with insomnia while relieving anxiety and depression and regulating neurotransmitter levels. However, there have been no meta-analyses on the effect of tuina on psychological states and neurotransmitter levels.

**Objectives:**

A meta-analysis was performed to systematically evaluate the effects of massage on sleep quality, psychological state, and neurotransmitter levels in patients with insomnia.

**Methods:**

A comprehensive literature search was conducted from inception to July 2023 using eight electronic databases to identify randomized controlled trials (RCTs) on tuina therapy for insomnia. Gray literature was also searched. The methodological quality of the included studies was assessed using the Cochrane Handbook. Reviewer Manager 5.4 and Stata 16.0 were employed for statistical analysis.

**Results:**

A total of 23 studies were included, including 1780 patients with insomnia, of whom 892 and 888 were in the experimental and control groups, respectively. Meta-analysis indicated that tuina therapy was superior to other therapies for the treatment of insomnia in increasing the total effective rate [OR = 4.12, 95%CI (2.80, 6.06), *p* < 0.00001] and 5-hydroxytryptamine (5-HT) level [MD = 16.03, 95% CI (13.40, 18.65), *p* < 0.00001], while reducing the Pittsburgh Sleep Quality Index score [MD = −2.34, 95% CI (−2.94, −1.74), *p* < 0.00001], Athens Insomnia Scale score [MD = −2.10, 95% CI (−2.67, −1.52), *p* < 0.00001], self-rating anxiety scale score [MD = −6.77, 95% CI (−8.34, −5.20), *p* < 0.00001] and self-rating depression scale score [MD = −6.60, 95% CI (−8.82, −4.37), *p* < 0.00001]. Subgroup analysis showed that tuina alone or in combination with other therapies was superior to drugs or acupuncture alone in improving all outcomes (*p* < 0.05). Only two studies reported minor adverse events.

**Conclusion:**

Tuina for insomnia has certain therapeutic advantages and can significantly improve sleep quality, relieve anxiety-depressive states, and increase 5-HT levels with high safety. Due to the limitations of the quality of the included studies, additional high-quality clinical trials are required for further verification.

**Systematic review registration:**

https://www.crd.york.ac.uk/PROSPERO/display_record.php?RecordID=447839, identifier CRD42023447839.

## Introduction

Insomnia is a common neurological disease, mainly characterized by difficulty falling asleep, difficulty maintaining sleep, and early waking (1, 2). With the increase in work pressure and accelerated pace of life, the incidence of insomnia is increasing annually (3). Chronic sleep deprivation can lead to emotional and mental problems, such as anxiety and depression. Recent studies have shown that the severity of anxiety and depression positively correlates with insomnia (4). The mechanism of insomnia is complex and related to a variety of neurotransmitters. Modern research generally believes that an abnormal level or reduced function of monoamine neurotransmitters is the biological basis of insomnia. 5-Hydroxytryptamine (5-HT), a representative monoamine neurotransmitter, is involved in the regulation of sleep activity and is closely associated with the occurrence and development of insomnia (5). Clinical treatment of insomnia is often with drugs, including benzodiazepines, non-benzodiazepines, antihistamines, and barbiturates. The efficacy of these drugs is reasonable, but there are many adverse reactions such as headache, vertigo, arrhythmia, and bleeding lesions, which are often intolerable by patients (6, 7). Therefore, it is necessary to develop more effective, stable, and safe treatments.

As a traditional Chinese external therapy, tuina has the advantages of simplicity, greenness, and safety, and has been widely used in the treatment of insomnia. Tuina is a treatment based on traditional Chinese medicine (TCM) Zang-Fu organ and meridian theories, and integrates modern scientific knowledge (such as biomechanical function, anatomy, pathology, and physiology) with traditional practice (8). It is a manual therapy that features a series of manipulations of the body, including pressing, rubbing, pulling, and pinching of soft tissues and sometimes rotating or manipulating extremities. Tuina treatment can regulate the qi and blood of the zang-fu organs, balance yin and yang in the body, and restore disorder, thereby improving sleep quality and extending sleep duration (9). Multiple meta-analyses (10, 11) have demonstrated that tuina, as a therapeutic measure for insomnia, has certain advantages over Chinese and Western medicine and acupuncture in terms of improving clinical efficiency and sleep quality. However, previous studies have failed to analyze the psychological states and neurotransmitter changes that affect insomnia. Therefore, this study applied a meta-analysis methodology to quantitatively evaluate the efficacy and safety of tuina therapy for insomnia and evaluated and analyzed other related factors (anxiety and depression state and neurotransmitter level) affecting the efficacy, aiming to provide more comprehensive and objective evidence-based medical support for the treatment of insomnia.

## Methods

### Trial registration

This study was conducted in accordance with the Preferred Reporting Items for Systematic Reviews and Meta-Analyses (PRISMA) statement (12). The protocol is registered in PROSPERO (registration number: CRD42023447839).

### Literature search

Online searches were conducted using Embase, Cochrane Library, PubMed, Web of Science, VIP Chinese Science and Technology Periodicals Full-Text (VIP), China National Knowledge Infrastructure (CNKI), Chinese Biomedical (CBM), and Wanfang databases. We also searched the gray literature and reviewed the reference lists of the included studies and related systematic reviews. Literature was searched from the date of the establishment of the database to July 5, 2023, in any language, and the search strategy was a combination of subject terms and free words. An example of the PubMed search strategy is provided in Supplementary Figure S1.

### Inclusion criteria

#### Type of study

Randomized controlled trials (RCTs) published at any time and in any language.

#### Types of participants

Patients with a clear diagnosis of insomnia based on the criteria developed by national or international authoritative organizations (13–16), regardless of age, race, or sex.

#### Types of interventions

The control group used only oral medicine or acupuncture alone, and the intervention measures of the experimental group were tuina alone, tuina combined with drugs, or tuina combined with acupuncture. The drug selection, dosage and frequency in the experimental and control groups in the same study should be consistent. To reduce potential bias and confounders due to differences in drugs, only benzodiazepines and non-benzodiazepines were included in this study.

#### Types of outcomes

Sleep quality indicators: Total effective rate, referring to the efficacy standards formulated by the State Administration of Traditional Chinese Medicine and authoritative experts (13, 14). The total effective rate was calculated as follows: = [(cure + marked effect + effective) number of cases ÷ total number of cases] × 100%; and the Pittsburgh Sleep Quality Index (PSQI) and Athens Insomnia Scale (AIS). Psychological state indicators: Self-rating anxiety scale (SAS) and self-rating depression scale (SDS). Neurotransmitter indicators: 5-hydroxytryptamine (5-HT).

### Exclusion criteria

The study types were as follows: reviews, cluster RCTs, animal experiments, expert experience, or case–control studies. Studies where diagnostic assessment criteria were not mentioned, duplicate publications, and literature with obvious statistical errors were excluded.

### Data extraction

Two reviewers independently extracted the information using an advance-designed standardized data extraction form. If a problem existed, it was resolved by a third party. The extracted information included the authors of the included studies, publication date, methodological design, sample size, disease duration, sex, age, intervention measures, treatment course, and outcome indicators.

### Risk of bias assessment

The risk of bias in the literature was evaluated by two investigators according to the risk of bias assessment tool in the Cochrane Reviewers Handbook 6.1.0 (17), including the following seven aspects: random sequence generation, allocation concealment, implementation of blinding method for patients and trial personnel, implementation of blinding method for outcome assessors, incomplete result data, selective reporting, and other biases (such as potential bias related to special study design and false statements). Ultimately, it was necessary to make a judgment on “low risk,” “high risk,” and “unclear risk” in the literature. Disagreements were resolved through a team discussion.

### Quality of evidence

The Grading of Recommendations Assessment, Development, and Evaluation (GRADE) approach was used to evaluate the quality of evidence for the primary outcomes and was categorized as high, moderate, low, or very low (18). Two authors without conflicts of interest related to this study reviewed the synthesized evidence and downgraded its certainty based on the study design, risk of bias, inconsistency, indirectness, and imprecision.

### Statistical analysis

Statistical analyses were performed using the Stata 16.0 and Review Manager 5.4 software. Heterogeneity among trials was assessed using Cochrane’s Q test and I-squared statistic (19). When *I*^2^ < 50%, *p* ≥ 0.1, it means that the heterogeneity among studies is small, and the fixed effect model is used for analysis; If *I*^2^ ≥ 50%, *p* < 0.1, the random effect model is used (20). The odds ratio (OR) was used as the effect size for dichotomous variables and the mean difference (MD) was used as the effect size for continuous variables to calculate the 95% confidence interval (CI). Furthermore, sensitivity analyses were performed to assess the stability of the results by deleting one study at a time, whereas subgroup analyses were performed based on different interventions. Publication bias was evaluated using funnel plots, Begg’s test, and Egger’s test (21). Sensitivity analysis and publication bias tests were performed only for outcome indicators in more than 10 articles.

## Results

### Study selection

**Figure 1:**
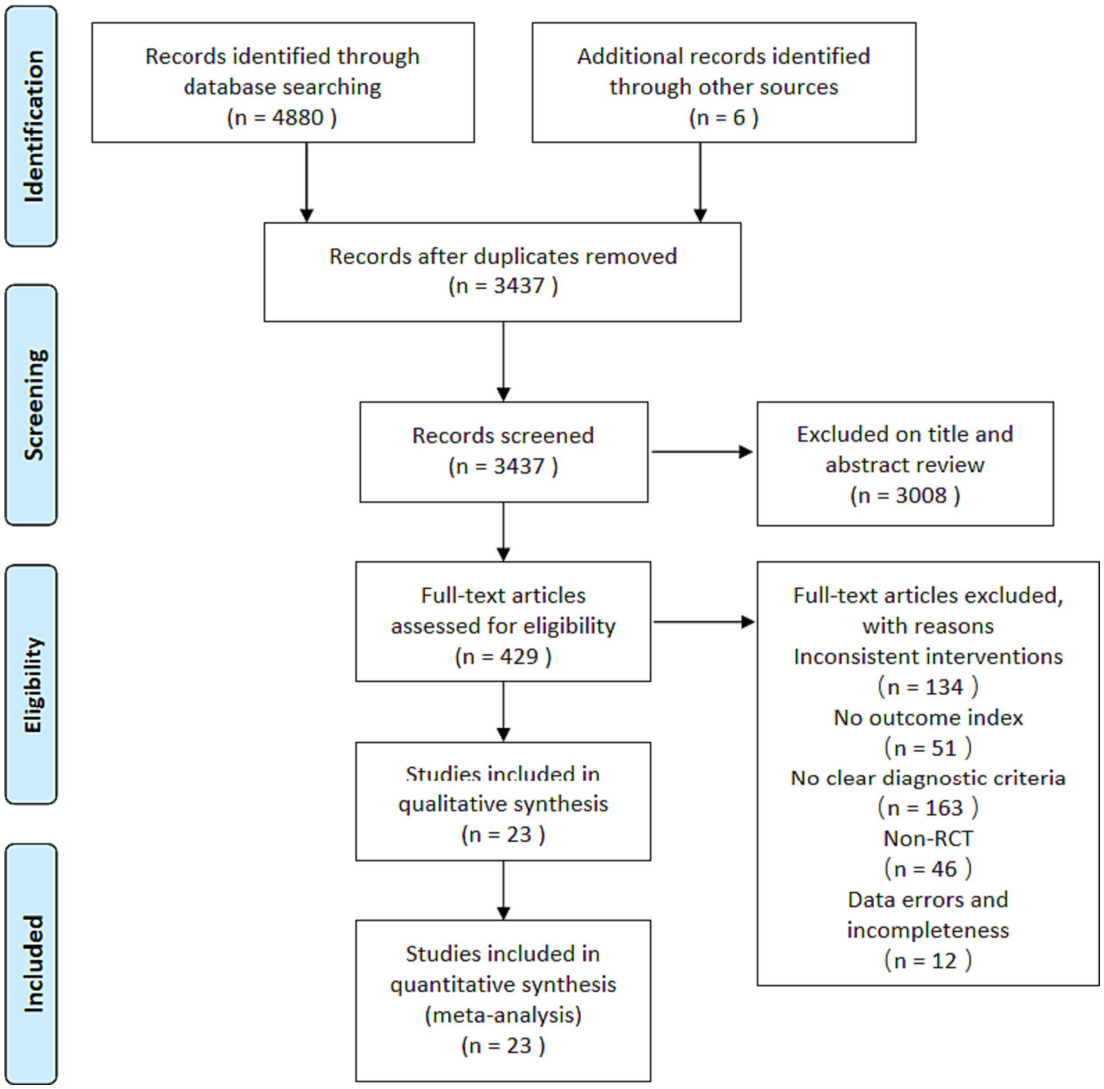
Literature search process.

A total of 4,886 articles were retrieved from the initial search, of which 4,880 were from databases and 6 were from other sources. Among the literatures retrieved in the database, 1,425 were from CNKI, 1163 VIP, 1269 Wanfang, 847 CBM, 21 Embase, 47 Cochrane Library, 72 PubMed, and 36 Web of Science. After using Endnote software to delete duplicate documents, 3,437 articles remained. After reading the titles and abstracts, 3,008 articles were excluded, leaving 429 articles. After reading the full text of these 429 articles, 406 articles that did not meet the inclusion criteria were excluded: 134 were inconsistent interventions, 163 did not have clear diagnostic criteria, 46 were not RCTs, 51 did not mention the outcome measures of this study, and 12 had obvious statistical errors. Finally, 23 studies (22–44) were qualified and included in this meta-analysis. A flowchart of the literature retrieval process is shown in Figure 1.

### Study characteristics

Twenty-three RCTs (22–44) were included in this study, of which one was in English (23) and the rest were in Chinese. A total of 1780 patients were included in the study, of whom 892 and 888 patients in the experimental and control groups, respectively, were diagnosed with insomnia. Two studies (23, 24) were multicenter trials and the rest were single-center studies. The sample sizes of the included studies ranged from 40 to 176. The intervention duration ranged from 2 to 8 weeks. Additional details of the included studies are presented in Table 1.

**Table 1:** Basic characteristics of the included studies.

Included studies	Mean age/years		Sample size (M/L)		Mean disease duration/year		Interventions		Duration/month	Outcome measures
	E	C	E	C	E	C	E	C		
Zhong ZG 2008 (22)	39.33 ± 13.52	34.10 ± 13.20	15/17	12/14	34.10 ± 13.20 mon	37.21 ± 11.53 mon	Tuina; Acupuncture; Alprazolam	Alprazolam	40 d	①②④⑤
Zhou YF 2008 (23)	42.30 ± 12.12	38.47 ± 11.99	84	82	–	–	Tuina	Guipi Pill	15 d	①④
Wang YX 2016 (24)	18 ~ 65	18 ~ 65	87	89	–	–	Tuina	Guipi Pill	15 d	①⑤
Gao SY 2020 (25)	43.03 ± 2.54	43.19 ± 2.63	32/22	30/24	3.59 ± 1.22	3.72 ± 1.38	Tuina; Acupuncture	Esazolam	30 d	①②④⑤
Wu MH 2019 (26)	35.43 ± 8.18	36.27 ± 9.45	13/17	11/19	3 ~ 82 mon	3 ~ 82 mon	Tuina	Guipi Decoction	4 weeks	①④
Tan ZY 2017 (27)	31.70 ± 12.65	38.33 ± 11.38	17/13	16/14	12.90 ± 6.79 w	12.03 ± 6.11 w	Tuina	Anshen Buxin Pill	1 month	①②④
Pan LK 2018 (28)	44.33 ± 10.37	47.03 ± 10.68	5/25	4/26	5.01 ± 4.42	5.43 ± 5.16	Tuina	Esazolam	2 weeks	①②④⑤
Fu LM 2016 (29)	47.7 ± 7.54	48.0 ± 5.99	17/13	14/16	16.37 ± 7.63 mon	15.93 ± 9.12 mon	Tuina	Zopiclone	4 weeks	④⑤
Zhang HS 2019 (30)	57.63 ± 3.90	57.20 ± 4.99	8/22	9/21	–	–	Tuina	Renshen Guipi Pill	4 weeks	①②③
Li QB 2021 (31)	51.4 ± 9.7	49.9 ± 12.5	15/15	16/14	29.0 ± 20.0	26.5 ± 16.8	Tuina; Acupuncture	Dexzopiclone	10 d	①②③
Chen SJ 2016 (32)	43.13 ± 5.05	44.52 ± 3.66	14/16	14/17	5.70 ± 1.39 mon	5.45 ± 1.48 mon	Tuina	Acupuncture	8 weeks	③
Li QB 2019 (33)	44.12 ± 18.24	41.42 ± 16.68	12/11	12/12	20.49 ± 50.26 d	19.25 ± 48.32 d	Tuina	Dexzopiclone	30 d	①③
Zhang Y 2022 (34)	48.90 ± 4.13	50.95 ± 4.90	4/16	6/14	-	-	Tuina	Renshen Guipi Pill	4 weeks	②③
Wei M 2017 (35)	47.40 ± 12.97	47.65 ± 12.60	16/13	12/18	26.20 ± 13.82 mon	26.40 ± 13.42 mon	Tuina	Esazolam	2 weeks	③
Wang J 2021 (36)	40.21 ± 3.94	40.19 ± 3.85	20/19	18/21	3.15 ± 0.56	3.11 ± 0.54	Tuina; Esazolam	Esazolam	2 months	⑥
Chen C 2021 (37)	52.32 ± 7.82	51.87 ± 7.71	33/27	31/29	–	–	Tuina; Acupuncture	Esazolam	2 weeks	①②④⑤⑥
Zhang HS 2020 (38)	51.30 ± 5.07	51.95 ± 4.66	3/17	5/15	–	–	Tuina	Renshen Guipi Pill	4 weeks	②③
Huang JJ 2020 (39)	36.42 ± 6.15	35.86 ± 6.65	32/18	30/20	3 mon~10 yr	3 mon~10 yr	Tuina	Esazolam	14 d	⑥
Zhang TY 2021 (40)	47.64 ± 5.78	45.19 ± 5.25	12/18	14/16	6.53 ± 1.26 mon	6.87 ± 1.14 mon	Tuina; Acupuncture	Esazolam	4 weeks	②④⑤⑥
Zhao Y 2013 (41)	37 ~ 48	37 ~ 48	30	30	–	–	Tuina	Esazolam	20 d	②⑥
Zang P 2023 (42)	42.88 ± 6.47	42.24 ± 5.78	35/29	32/31	10.39 ± 1.67 mon	10.83 ± 1.58 mon	Tuina; Acupuncture; Esazolam	Esazolam	15 d	①②⑥
Tan T 2014 (43)	39.6 ± 8.0	37.3 ± 10.0	18/12	20/10	–	–	Tuina	Esazolam	20 d	①⑥
Lou HJ 2018 (44)	37.63 ± 3.90	37.20 ± 4.99	14/16	15/15	–	–	Tuina	Renshen Guipi Pill	4 weeks	①②③

E, Experimental group; C, Control group; ①, Total effective rate; ②, PSQI; ③, AIS; ④, SAS; ⑤, SDS; ⑥, 5-HT.

### Bias and GRADE assessment

**Figure 2:**
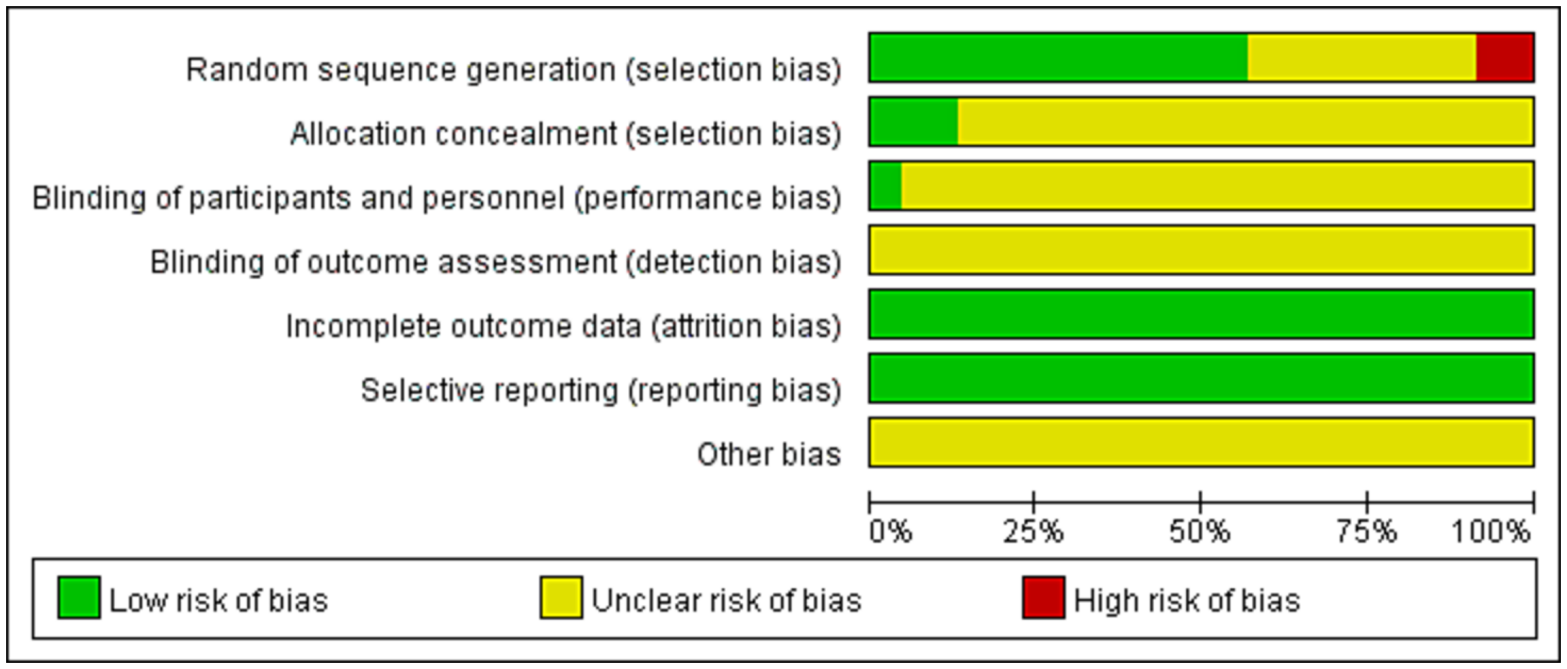
Literature bias assessment results.

Randomization was mentioned in all studies. Eleven (26, 27, 29, 30, 33, 34, 37–40, 43) used the random number table method and two (42, 44) used the envelope method and were assessed as low risk, two (22, 28) randomized according to the order of attendance and were assessed as high risk, and the remaining eight did not describe the specific method of randomization. Only one study (36) mentioned the use of blinding, and three studies (26, 42, 44) involved allocation concealment to be assessed as low risk, while the remaining studies did not mention allocation concealment and blinding. All 23 studies reported the outcome indicators used in this study, and none identified falsified or incomplete data, with incomplete reporting and early discontinuation of trials. No other biases were mentioned in any of the studies. The results are shown in Figure 2, and a summary of the risk of bias is shown in Supplementary Figure S2. The GRADE results are shown in Supplementary Figure S3. In terms of importance, other outcome indicators were important in addition to the total effective rate and PSQI. Moreover, the quality of evidence was very low or low because of poor methodological quality.

## Results of meta-analysis

### Sleep quality

#### Total effective rate

**Figure 3:**
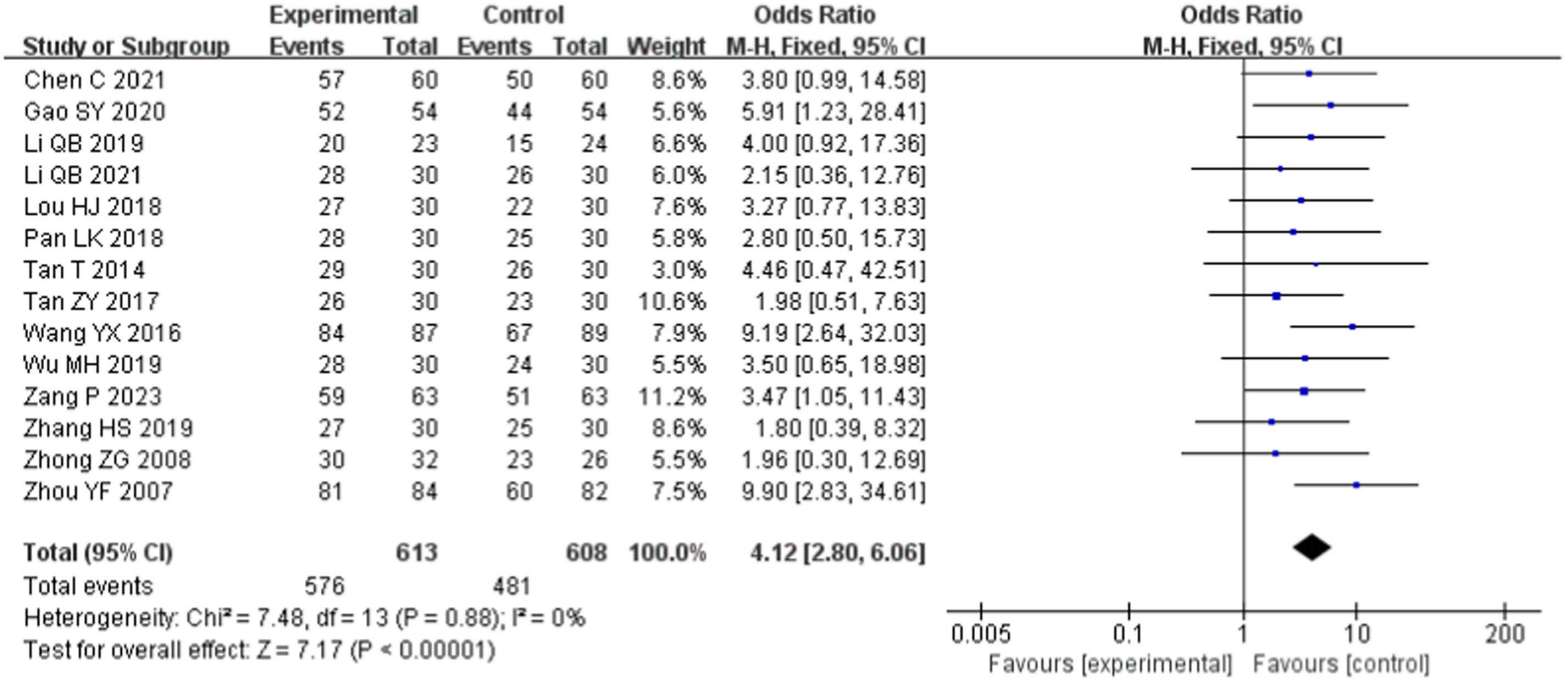
Meta-analysis of total effective rate.

Fourteen papers (22–28, 30, 31, 33, 37, 42–44) reported the total effective rate and included 1,221 patients: 613 in the experimental group and 608 in the control group. The combined analysis showed no heterogeneity between studies (*p* = 0.88, *I*^2^ = 0) and thus was analyzed using a fixed-effects model. The results showed a statistically significant difference [OR = 4.12, 95% CI (2.80, 6.06), *p* < 0.00001; Figure 3], suggesting a higher overall effectiveness rate of tuina for insomnia.

**Figure 4:**
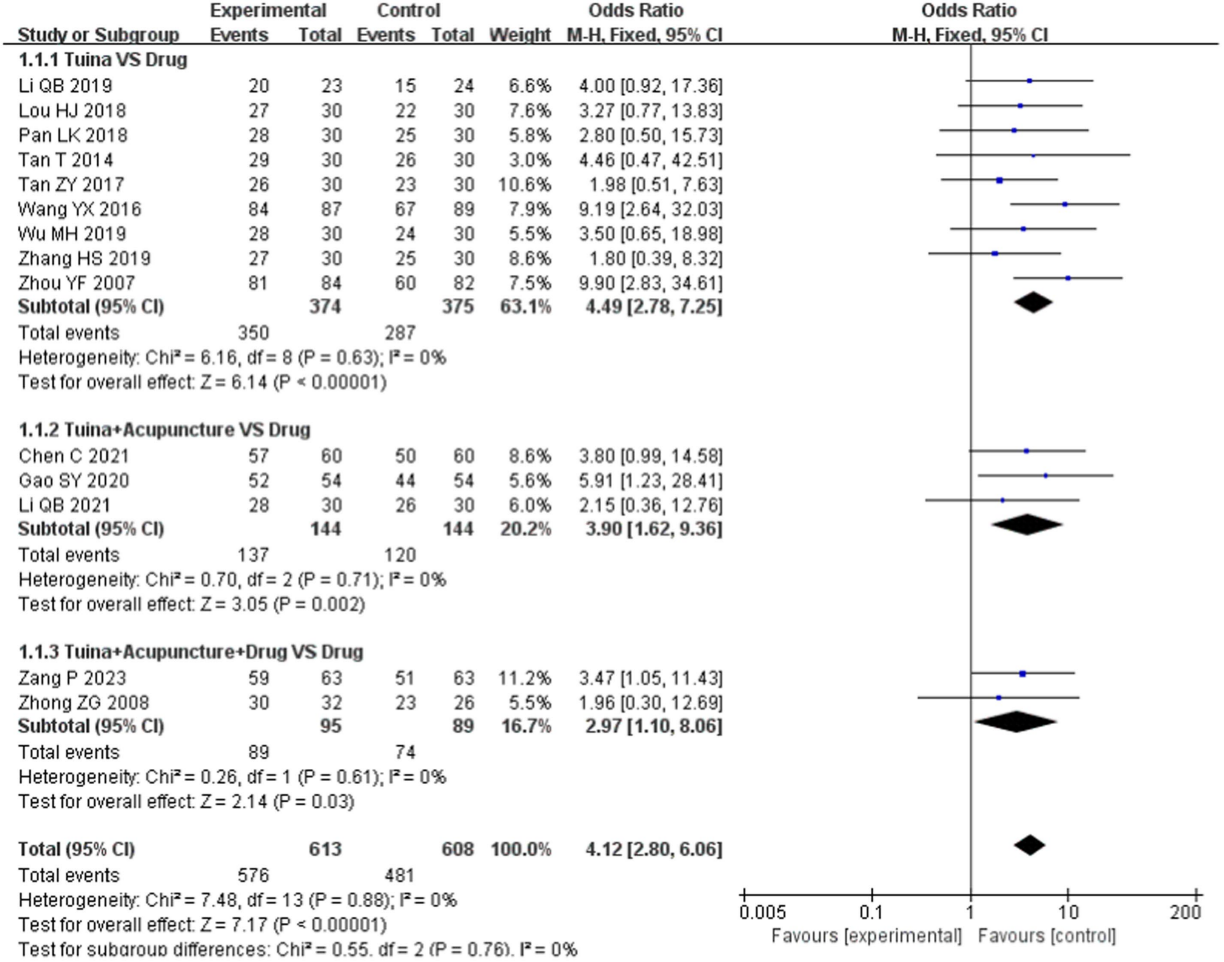
Subgroup analysis of total effective rate.

In the subgroup analysis, as an independent treatment, tuina therapy improved insomnia more than drug therapy [OR = 4.49, 95% CI (2.78, 7.25), *p* < 0.00001; Figure 3]. Compared with drugs, tuina+acupuncture and tuina+acupuncture+drugs significantly improved the total effective rate (*p* < 0.05), and there was no statistically significant difference between the subgroups (*p* = 0.76, *I*^2^ = 0), as shown in Figure 4.

#### PSQI

**Figure 5:**
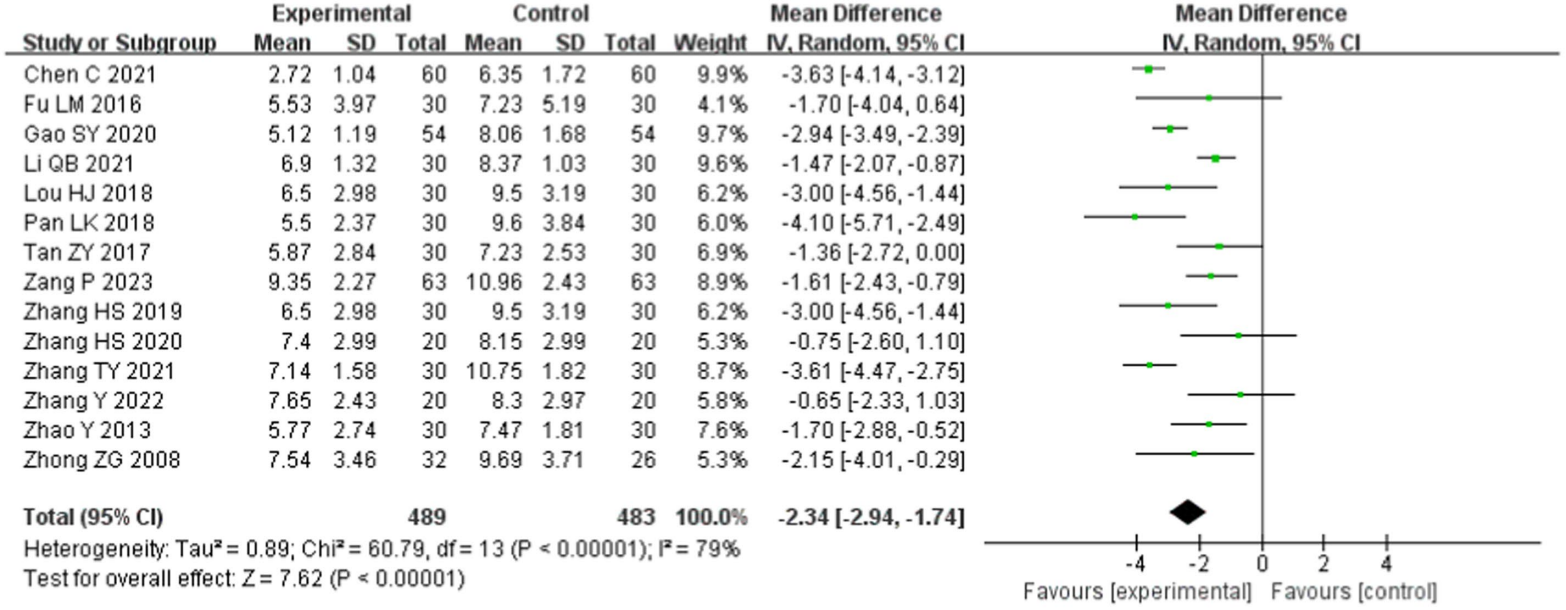
Meta-analysis of PSQI.

Fourteen studies (22, 25, 27, 28, 30, 31, 34, 37–41, 44) mentioned the PSQI, including 972 patients: 489 in the experimental group and 483 in the control group. There was significant heterogeneity among the studies (*p* < 0.00001, *I*^2^ = 79%), which was analyzed using a random-effects model. The results showed that the difference was statistically significant [MD = −2.34, 95% CI (−2.94, −1.74), *p* < 0.00001; Figure 5], indicating that tuina had a greater effect on reducing the PSQI score.

**Figure 6:**
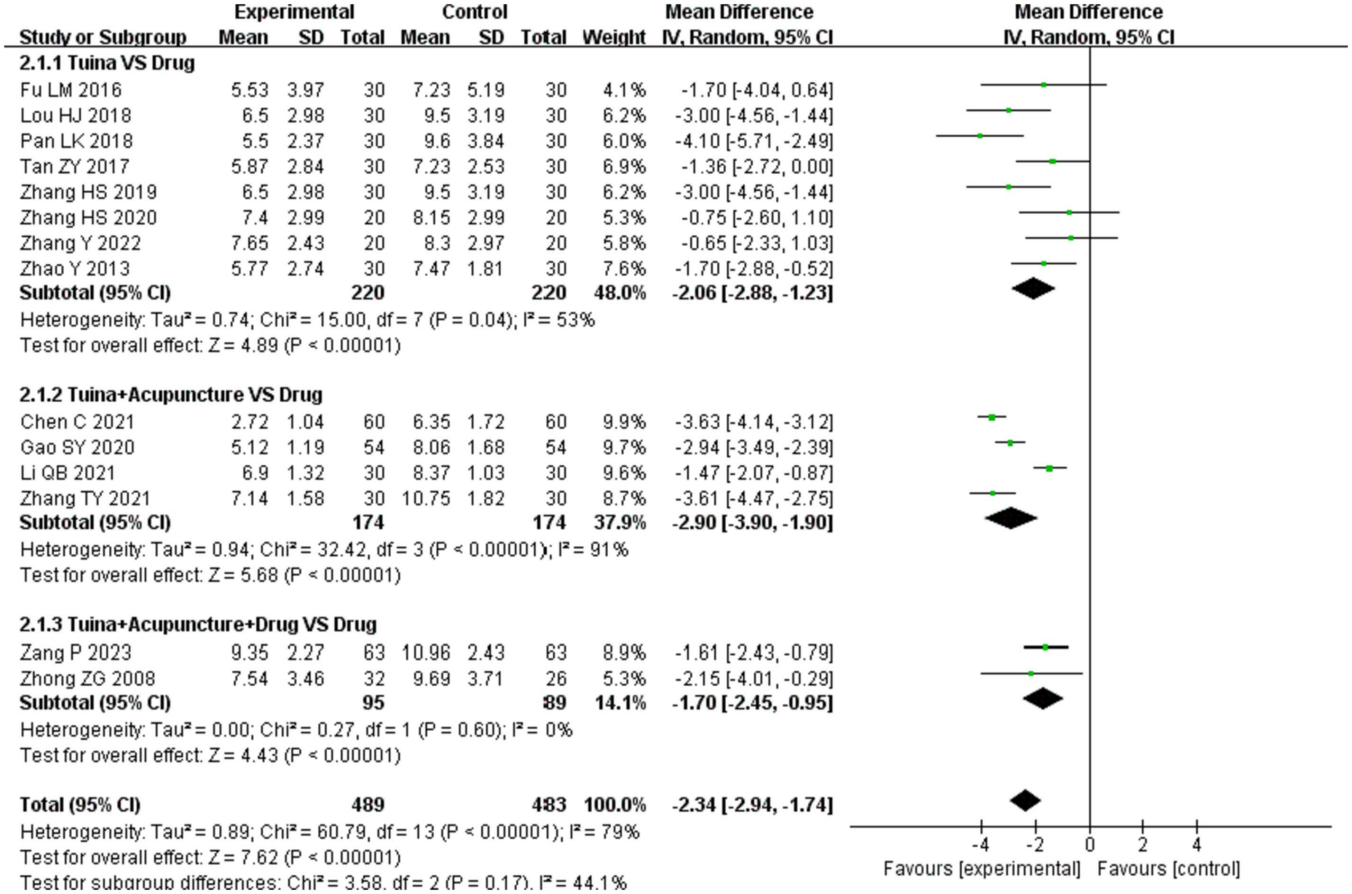
Subgroup analysis of PSQI.

Subgroup analyses based on different interventions showed that as an independent therapy, tuina therapy improved insomnia more than drug therapy [MD = −2.06, 95% CI (−2.88, −1.23), *p* < 0.00001; Figure 5]. Compared to drugs, tuina+acupuncture and tuina+acupuncture+drugs significantly reduced the PSQI score (*p* < 0.05). The differences between the subgroups were small and not statistically significant (*p* = 0.17, *I*^2^ = 44.1%), as shown in Figure 6.

#### AIS

Eight studies (30–35, 38, 44) mentioned AIS, including 428 patients, with 213 in the experimental group and 215 in the control group. There was some heterogeneity among the studies (*p* = 0.02, *I*^2^ = 57%), which was analyzed using a random-effects model. The results showed that the difference was statistically significant [MD = −2.10, 95% CI (−2.67, −1.52), *p* < 0.00001], indicating that tuina had a greater effect in reducing the AIS score, as shown in Supplementary Figure S4.

Subgroup analysis according to different interventions showed that tuina could better reduce the AIS score than acupuncture [MD = −1.80, 95% CI (−2.61, −0.99), *p* < 0.0001]. Compared with oral drugs, tuina alone or in combination with acupuncture reduced the AIS score to a certain extent, and the difference was statistically significant (*p* < 0.05) (Supplementary Figure S5). The combined analysis of the two subgroups whose control group was drug therapy, showed that the difference between the two subgroups was statistically significant (*p* = 0.006, *I*^2^ = 87.0%). Tuina combined with acupuncture was superior to tuina alone in improving AIS scores (Supplementary Figure S6).

### Psychological state

#### SAS

SAS scores were reported in nine studies (22, 23, 25–29, 37, 40) involving 752 patients, including 380 patients in the experimental group and 372 patients in the control group. Due to the large heterogeneity among the studies (*p* = 0.0002, *I*^2^ = 73%), a random-effects model was adopted. The results showed that the difference was statistically significant [MD = −6.77, 95% CI (−8.34, −5.20), *p* < 0.00001], suggesting that tuina treatment in patients with insomnia can significantly reduce SAS scores (Supplementary Figure S7).

Subgroup analysis was conducted according to different interventions, and the results showed that compared with oral drugs, tuina, tuina+acupuncture, and tuina+acupuncture+drug significantly reduced the SAS score, with statistical differences (*p* < 0.05); there was no difference among subgroups (*p* = 0.44, *I*^2^ = 0) (Supplementary Figure S8).

#### SDS

Seven articles (22, 24, 25, 28, 29, 37, 40) included the SDS scores of 642 patients, including 323 in the experimental group and 319 in the control group. Owing to the large heterogeneity among the studies (*p* < 0.0001, *I*^2^ = 79%), a random-effects model was used. The results showed that the difference was statistically significant [MD = −6.60, 95% CI (−8.82, −4.37), *p* < 0.00001], indicating that tuina had a greater effect on reducing the SDS score (Supplementary Figure S9).

Subgroup analysis according to different intervention measures showed that compared with oral drugs, tuina and tuina+acupuncture could significantly reduce the SDS score (*p* < 0.05), and there was no significant difference in SDS score reduction between tuina+acupuncture+drug and oral drug (*p* = 0.06). There were no significant differences between the subgroups (*p* = 0.67, *I*^2^ = 0) (Supplementary Figure S10).

### Neurotransmitter level

#### 5-HT

Seven studies (36, 37, 39–43) reported on 5-HT involving 604 patients: 302 in the experimental group and 302 in the control group. The combined analysis showed some heterogeneity among the studies (*p* = 0.006, *I*^2^ = 67%), which were analyzed using a random-effects model. The results showed a statistically significant difference [MD = 16.03, 95% CI (13.40, 18.65), *p* < 0.00001], suggesting that tuina has a greater effect in increasing 5-HT levels (Supplementary Figure S11).

In the subgroup analysis, as an independent treatment, tuina therapy improved 5-HT levels more than drug therapy [MD = 17.82, 95% CI (11.58, 24.05), *p* < 0.00001]. Compared with drugs, tuina+acupuncture, tuina+drugs and tuina+acupuncture+drugs significantly improved 5-HT levels (*p* < 0.05), with no statistically significant difference between subgroups (*p* = 0.60, *I*^2^ = 0) (Supplementary Figure S12).

### Adverse reaction

Only two studies (36, 42) mentioned the occurrence of adverse reactions, the incidence of which was 12.82% in the experimental group and 10.26% in the control group, and 7.9% in the experimental group and 9.5% in the control group, respectively. All of them were mild, mainly manifested as drowsiness, itchy skin, and malaise. No serious adverse reactions were observed.

### Sensitivity analysis

To further confirm the stability of the meta-analysis results, a sensitivity analysis was conducted by excluding studies from the analysis individually. We only analyzed the outcome indicators of more than 10 studies, including the total effective rate and PSQI. The results showed that the pooled effect of tuina on the above outcome measures did not change significantly when individual studies were omitted (Supplementary Figures S13, S14).

### Publication bias

**Figure 7:**
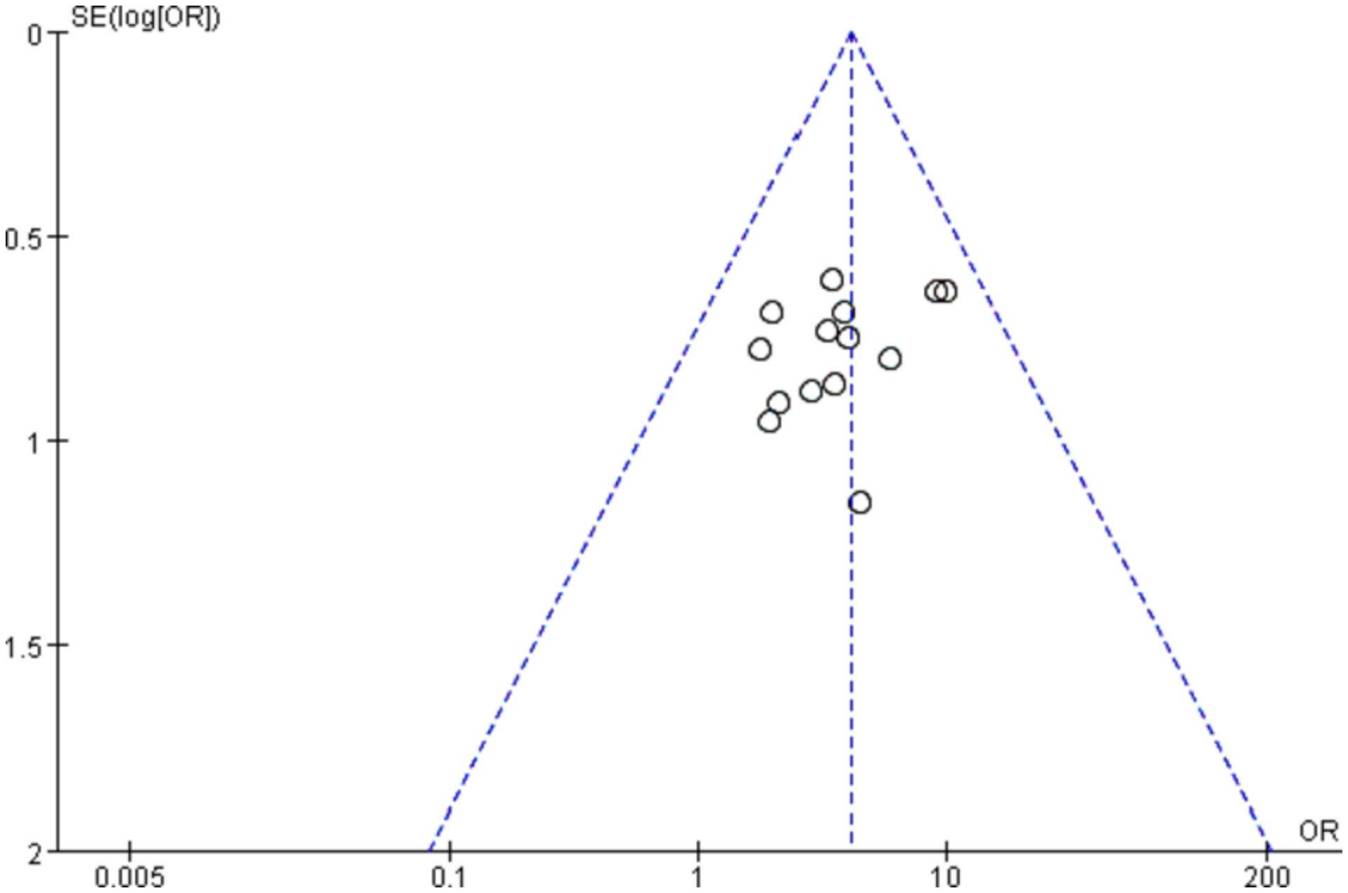
Funnel plot of total effective rate.

**Figure 8:**
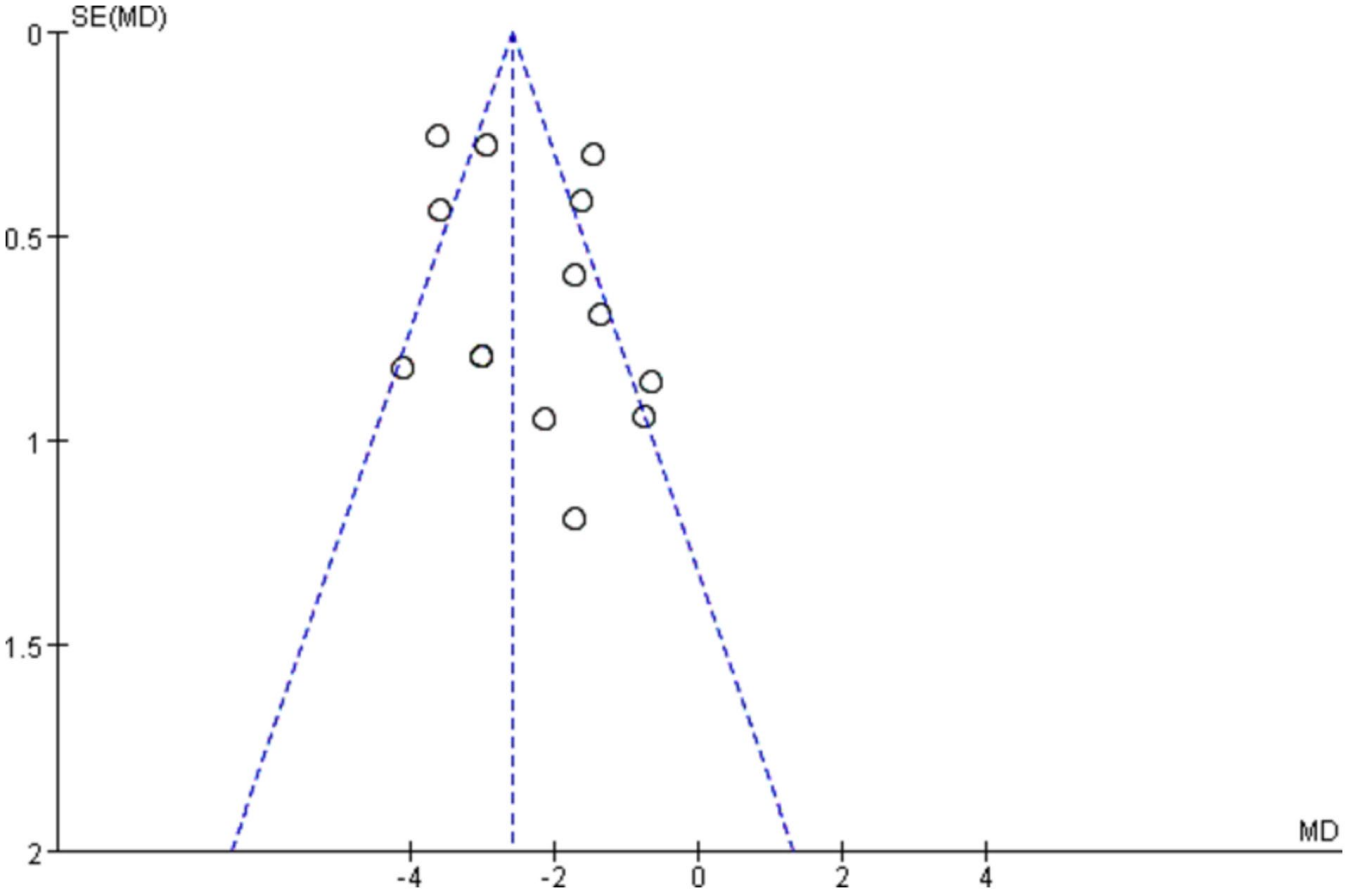
Funnel plot of PSQI.

The shape of the funnel plots (total effective rate and PSQI) did not show visual evidence of asymmetry, indicating few possible publication biases (Figures 7, 8). Neither Begg’s test nor Egger’s test detected a significant publication bias (*p* > 0.05) (Supplementary Table S1).

## Discussion

In recent years, tuina has shown unique advantages in the treatment of insomnia and related diseases, attracting increasing attention from clinicians and patients. Several guidelines and consensus list tuina as a common therapeutic measure for the treatment of insomnia (45, 46). In this study, the latest evidence in the literature was included to investigate the effects of tuina on anxiety and depression status and 5-HT levels in patients with insomnia based on an analysis of sleep quality and its correlation, resulting in a meta-analysis of 23 papers.

The total effective rate, PSQI, and AIS are clinical trial indicators of sleep quality in patients with insomnia, and the results of these outcome indicators showed that tuina therapy can significantly improve sleep quality (*p* < 0.05). In this study, SAS and SDS were used as observation indexes of anxiety and depression status, and the study showed that tuina significantly alleviated patients’ anxiety and depression status (*p* < 0.05). In addition, tuina significantly increased the 5-HT content in patients (*p* < 0.05). Only two studies mentioned the occurrence of adverse reactions, mainly in the form of drowsiness, skin itching, and fatigue. No serious adverse reactions were observed, suggesting that tuina therapy is safe. Subgroup analyses based on different interventions showed that, except for the SDS score, the improvement in all indicators using tuina alone or in combination with other therapies was better than that using drugs or acupuncture alone (*p* < 0.05). There were no significant differences between the subgroups for the remaining indicators, except for the AIS score (*p* > 0.05), where acupuncture combined with tuina was superior to tuina alone in reducing the AIS score (*p* < 0.05).

Tuina acts on the human body and stimulates the patient’s acupoints through manipulation, which can regulate the whole body’s qi and blood and adjust the balance of yin and yang to achieve the effect of yin and yang secrecy, as well as the spirit and even the effect. Studies have shown that tuina can activate the corticotropin releasing hormone/corticotropin releasing hormone receptor 1 (CRH/CRHR1) pathway in the hypothalamus, activate the hypothalamic–pituitary–adrenal (HPA) axis, and regulate metabolites such as adrenocorticotropic hormone (ACTH), cortisol, 5-HT, and gamma-aminobutyric acid (GABA), thus achieving the purpose of treating insomnia (34). The relationship between insomnia, depression, and anxiety is bidirectional, and poor psychological status can affect the effectiveness of insomnia treatment (47). In this study, we found that the improvement of sleep disorder symptoms after tuina intervention was accompanied by the relief of anxiety and depression.

Several studies have demonstrated that 5-HT, as a monoamine neurotransmitter, and its related receptor function can regulate the sleep–wake rhythm, improve sleep structure, and excite the neurons of the nucleus accumbens to promote sleep (48, 49). At the same time, it can inhibit the central nervous system, stimulate autonomic reflexes, improve the function of the hippocampal glucocorticoid receptor-HPA axis, and cause the cerebral cortex to exert a mediating role in alleviating anxiety and depression. After a comprehensive analysis of this study, it was found that massage could not only alleviate patients’ sleep disorders but also effectively improve anxiety and depression states and 5-HT levels, which was consistent with the above studies. In terms of improving AIS scores, the effect of tuina combined with acupuncture was better than that of tuina alone, which may be related to the fact that acupuncture can improve the 5-HT_1A_R (5-HT_2A_R)/AC signaling pathway, promote 5-HT secretion, and regulate sleep-related neurons, which together with the combination of tuina, exerted a significant synergistic effect (50, 51).

This study had some limitations. (1) The quality of the research literature was uneven; only 15 out of 23 RCTs mentioned the random allocation method, and 4 studies mentioned allocation concealment and blinding, so there may be implementation bias. (2) Only two articles mentioned the occurrence of adverse reactions, therefore meta-analysis could not be performed and the conclusions obtained may have some bias. (3) Most studies did not mention follow-up, making it more difficult to judge long-term efficacy. (4) There are many subjective indices; however, only 5-HT is an objective index, and the results of the trial may be affected by subjectivity. (5) The sample size of the included studies was small; most were single-center trials, and only two were multicenter studies.

In summary, tuina treatment for insomnia is safe and effective and can improve sleep quality, alleviate anxiety and depression, increase 5-HT levels, with no obvious adverse reactions. In the clinical treatment process, it can be appropriately combined with acupuncture or medicine according to the individual situation. Owing to the limited number and quality of the included studies, the conclusions obtained need to be further verified.

## Data availability statement

The original contributions presented in the study are included in the article/Supplementary material, further inquiries can be directed to the corresponding author.

## Author contributions

ZhenW: Writing – original draft, Writing – review & editing, Methodology, Project administration. HX: Methodology, Writing – review & editing. ZhengW: Data curation, Software, Writing – review & editing. HZ: Data curation, Methodology, Software, Writing – review & editing. LZ: Data curation, Methodology, Project administration, Visualization, Writing – review & editing. YW: Data curation, Investigation, Methodology, Software, Writing – review & editing. ML: Resources, Software, Visualization Writing – review & editing. YZ: Writing – original draft, Writing – review & editing.
