# Supplementary material for: Effect of tuina on sleep quality, psychological state and neurotransmitter level in patients with insomnia: a systematic review and meta-analysis
Source: Front Neurol. 2024 Feb 21;15:1273194. doi: 10.3389/fneur.2024.1273194 (PMC10914942; doi:10.3389/fneur.2024.1273194)
Supplement: Supplementary file 1 [file Data_Sheet_1.PDF]

# Effect of Tuina on Sleep Quality, Psychological State and Neurotransmitter Level in Patients with Insomnia: A Systematic Review and Meta-Analysis

## Supplementary materials


| | | |
|---|---|---|
| **Search Strategies of Pubmed** | **Figure S1** | **Page 2** |
| **Risk of bias summary** | **Figure S2** | **Page 3** |
| **GRADE assessment of quality of evidence** | **Figure S3** | **Page 4** |
| **Results of meta-analysis** | **Figure S4-12** | **Page 5-8** |
| **Results of sensitivity analysis** | **Figure S13-14** | **Page 9** |
| **Begger's test and Egger's test** | **Table S1** | **Page 10** |

| Number | Search terms |
|---|---|
| 1 | exp Insomnia/Sleeplessness/Agrypnia |
| 2 | exp Insomnia disorder/DIMS |
| 3 | Sleep initiation and maintenance disorders .ti,ab |
| 4 | Early awakening .ti,ab |
| 5 | or 1-4 |
| 6 | Tuina .ti,ab |
| 7 | Massage .ti,ab |
| 8 | Massotherapy .ti,ab |
| 9 | Manipulation .ti,ab |
| 10 | or 6-9 |
| 11 | exp randomized controlled clinical trial .pt |
| 12 | exp controlled clinical trial .pt |
| 13 | randomised .ti,ab |
| 14 | randomized .ti,ab |
| 15 | randomly .ti,ab |
| 16 | trial .ti,ab |
| 17 | groups.ti,ab |
| 18 | or 11-17 |
| 19 | exp animals/not humans.sh. |
| 20 | 18 not 19 |
| 21 | 5 and 10 and 20 |

**Figure S1 The search strategies of Pubmed**

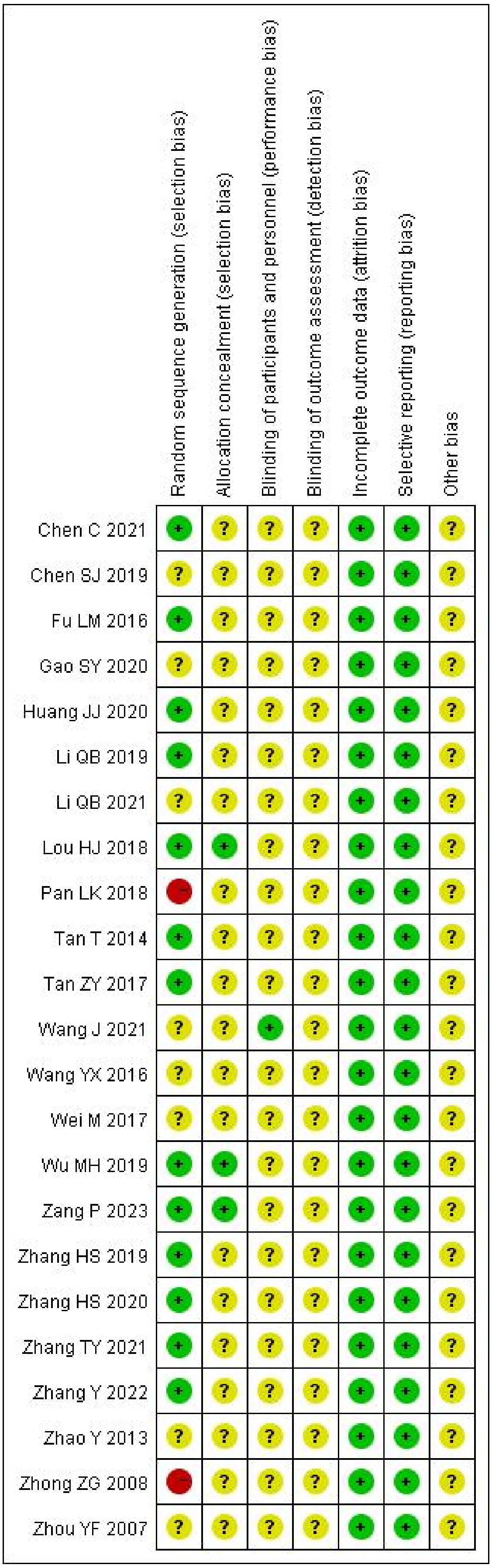


**Figure S2 Risk of bias summary**

| Certainty assessment | | | | | | | № of patients | | Effect | Certainty | Importance |
|---|---|---|---|---|---|---|---|---|---|---|---|
| № of studies | Study design | Risk of bias | Inconsistency | Indirectness | Imprecision | Other considerations | [patients] | [controls] | Absolute (95% CI) | | |
| **Total effective rate** | | | | | | | | | | | |
| 14 | observational studies | not serious | serious | not serious | not serious | none | 613 cases 608 controls | | - | ⊕◯◯◯ Very low | CRITICAL |
| | | | | | | | - | 0.0% | 4.12 (2.80 to 6.06) | | |
| **PSQI** | | | | | | | | | | | |
| 14 | observational studies | not serious | not serious | not serious | not serious | none | 489 cases 483 controls | | - | ⊕⊕◯◯ Low | CRITICAL |
| | | | | | | | - | 0.0% | -2.34 (-2.94 to -1.74) | | |
| **AIS** | | | | | | | | | | | |
| 8 | observational studies | not serious | not serious | serious | not serious | none | 213 cases 215 controls | | - | ⊕◯◯◯ Very low | IMPORTANT |
| | | | | | | | - | 0.0% | -2.10 (-2.67 to -1.52) | | |
| **SAS** | | | | | | | | | | | |
| 9 | observational studies | not serious | not serious | not serious | not serious | none | 380 cases 372 controls | | - | ⊕⊕◯◯ Low | IMPORTANT |
| | | | | | | | - | 0.0% | -6.77 (-8.34 to -5.20) | | |
| **SDS** | | | | | | | | | | | |
| 7 | observational studies | not serious | not serious | not serious | not serious | none | 323 cases 319 controls | | - | ⊕⊕◯◯ Low | IMPORTANT |
| | | | | | | | - | 0.0% | -6.60 (-8.82 to -4.37) | | |
| **5-HT** | | | | | | | | | | | |
| 7 | observational studies | not serious | serious | not serious | not serious | none | 302 cases 302 controls | | - | ⊕◯◯◯ Very low | IMPORTANT |
| | | | | | | | - | 0.0% | 16.03 (13.40 to 18.65) | | |

**Figure S3 GRADE assessment of quality of evidence**

# Results of Meta-Analysis

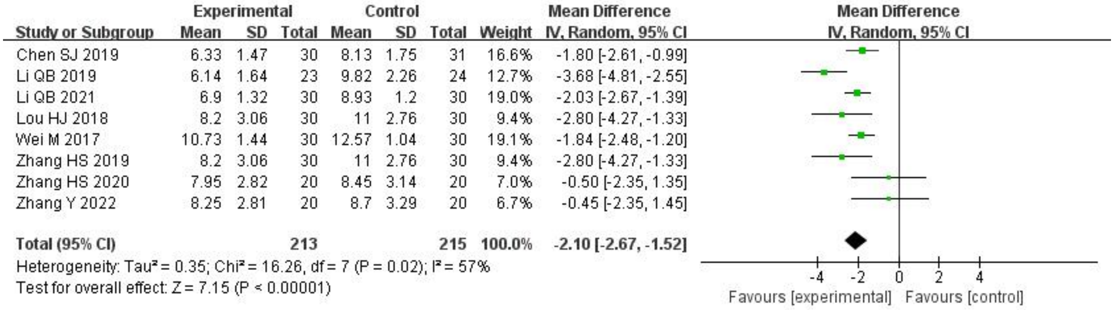


**Figure S4 Meta-analysis of AIS**

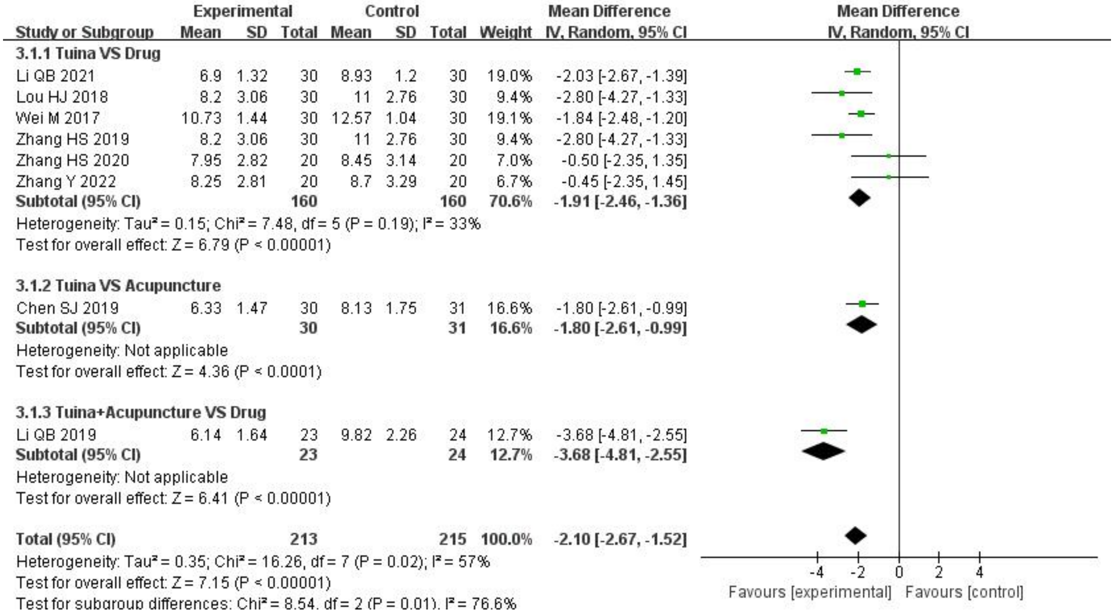


**Figure S5 Subgroup analysis of AIS**

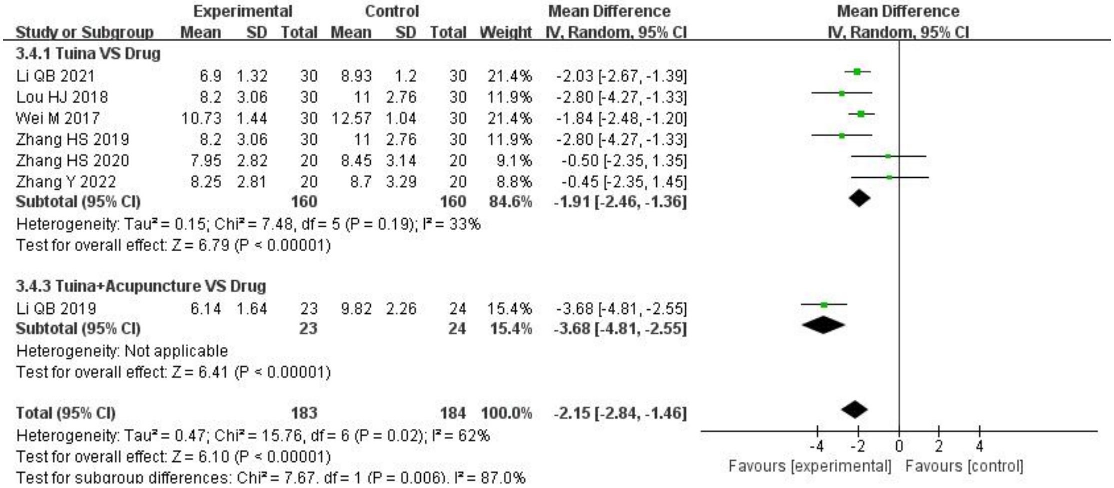


**Figure S6 Two subgroup analysis of AIS**

| Study or Subgroup | Experimental Mean | Experimental SD | Experimental Total | Control Mean | Control SD | Control Total | Weight | Mean Difference IV, Random, 95% CI |
|---|---|---|---|---|---|---|---|---|
| Chen C 2021 | 41.48 | 5.95 | 60 | 50.59 | 8.17 | 60 | 11.8% | -9.11 [-11.67, -6.55] |
| Fu LM 2016 | 44.94 | 8.3 | 30 | 53.03 | 10.94 | 30 | 6.4% | -8.09 [-13.00, -3.18] |
| Gao SY 2020 | 33.44 | 3.01 | 54 | 40.31 | 3.09 | 54 | 15.6% | -6.87 [-8.02, -5.72] |
| Pan LK 2018 | 34.58 | 5.09 | 30 | 39.29 | 7.56 | 30 | 9.8% | -4.71 [-7.97, -1.45] |
| Tan ZY 2017 | 30.77 | 3.83 | 30 | 37.47 | 3.04 | 30 | 14.1% | -6.70 [-8.45, -4.95] |
| Wu MH 2019 | 39.83 | 2.8 | 30 | 42.6 | 4.4 | 30 | 13.8% | -2.77 [-4.64, -0.90] |
| Zhang TY 2021 | 41.05 | 4.42 | 30 | 48.77 | 5.68 | 30 | 11.7% | -7.72 [-10.30, -5.14] |
| Zhong ZG 2008 | 40.52 | 6.51 | 32 | 45.64 | 7.52 | 26 | 8.9% | -5.12 [-8.79, -1.45] |
| Zhou YF 2007 | 42.74 | 12.99 | 84 | 54.84 | 13.84 | 82 | 7.9% | -12.10 [-16.19, -8.01] |
| **Total (95% CI)** | | | **380** | | | **372** | **100.0%** | **-6.77 [-8.34, -5.20]** |

Heterogeneity: Tau² = 3.77; Chi² = 29.98, df = 8 (P = 0.0002); I² = 73%
Test for overall effect: Z = 8.44 (P < 0.00001)

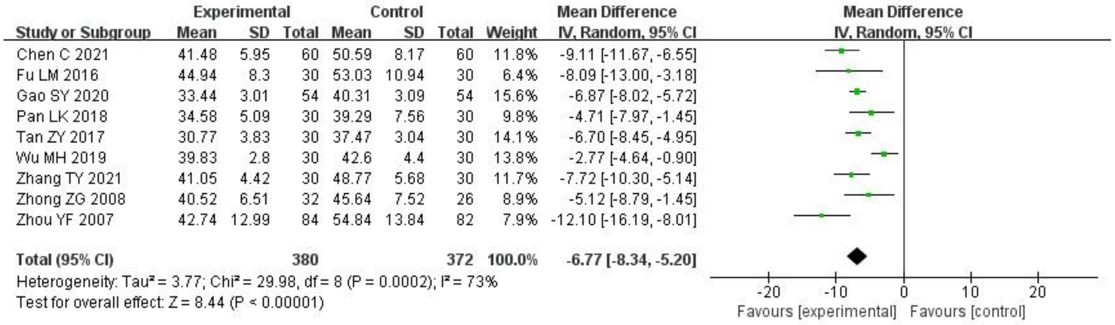


**Figure S7 Meta-analysis of SAS**

| Study or Subgroup | Experimental Mean | Experimental SD | Experimental Total | Control Mean | Control SD | Control Total | Weight | Mean Difference IV, Random, 95% CI |
|---|---|---|---|---|---|---|---|---|
| **4.1.1 Tuina VS Drug** | | | | | | | | |
| Fu LM 2016 | 44.94 | 8.3 | 30 | 53.03 | 10.94 | 30 | 6.4% | -8.09 [-13.00, -3.18] |
| Pan LK 2018 | 34.58 | 5.09 | 30 | 39.29 | 7.56 | 30 | 9.8% | -4.71 [-7.97, -1.45] |
| Tan ZY 2017 | 30.77 | 3.83 | 30 | 37.47 | 3.04 | 30 | 14.1% | -6.70 [-8.45, -4.95] |
| Wu MH 2019 | 39.83 | 2.8 | 30 | 42.6 | 4.4 | 30 | 13.8% | -2.77 [-4.64, -0.90] |
| Zhou YF 2007 | 42.74 | 12.99 | 84 | 54.84 | 13.84 | 82 | 7.9% | -12.10 [-16.19, -8.01] |
| **Subtotal (95% CI)** | | | **204** | | | **202** | **52.0%** | **-6.51 [-9.38, -3.64]** |
| Heterogeneity: Tau² = 8.09; Chi² = 21.34, df = 4 (P = 0.0003); I² = 81% | | | | | | | | |
| Test for overall effect: Z = 4.44 (P < 0.00001) | | | | | | | | |
| **4.1.2 Tuina+Acupuncture VS Drug** | | | | | | | | |
| Chen C 2021 | 41.48 | 5.95 | 60 | 50.59 | 8.17 | 60 | 11.8% | -9.11 [-11.67, -6.55] |
| Gao SY 2020 | 33.44 | 3.01 | 54 | 40.31 | 3.09 | 54 | 15.6% | -6.87 [-8.02, -5.72] |
| Zhang TY 2021 | 41.05 | 4.42 | 30 | 48.77 | 5.68 | 30 | 11.7% | -7.72 [-10.30, -5.14] |
| **Subtotal (95% CI)** | | | **144** | | | **144** | **39.1%** | **-7.48 [-8.71, -6.24]** |
| Heterogeneity: Tau² = 0.31; Chi² = 2.56, df = 2 (P = 0.28); I² = 22% | | | | | | | | |
| Test for overall effect: Z = 11.88 (P < 0.00001) | | | | | | | | |
| **4.1.3 Tuina+Acupuncture+Drug VS Drug** | | | | | | | | |
| Zhong ZG 2008 | 40.52 | 6.51 | 32 | 45.64 | 7.52 | 26 | 8.9% | -5.12 [-8.79, -1.45] |
| **Subtotal (95% CI)** | | | **32** | | | **26** | **8.9%** | **-5.12 [-8.79, -1.45]** |
| Heterogeneity: Not applicable | | | | | | | | |
| Test for overall effect: Z = 2.74 (P = 0.006) | | | | | | | | |
| **Total (95% CI)** | | | **380** | | | **372** | **100.0%** | **-6.77 [-8.34, -5.20]** |

Heterogeneity: Tau² = 3.77; Chi² = 29.98, df = 8 (P = 0.0002); I² = 73%
Test for overall effect: Z = 8.44 (P < 0.00001)
Test for subgroup differences: Chi² = 1.64, df = 2 (P = 0.44), I² = 0%

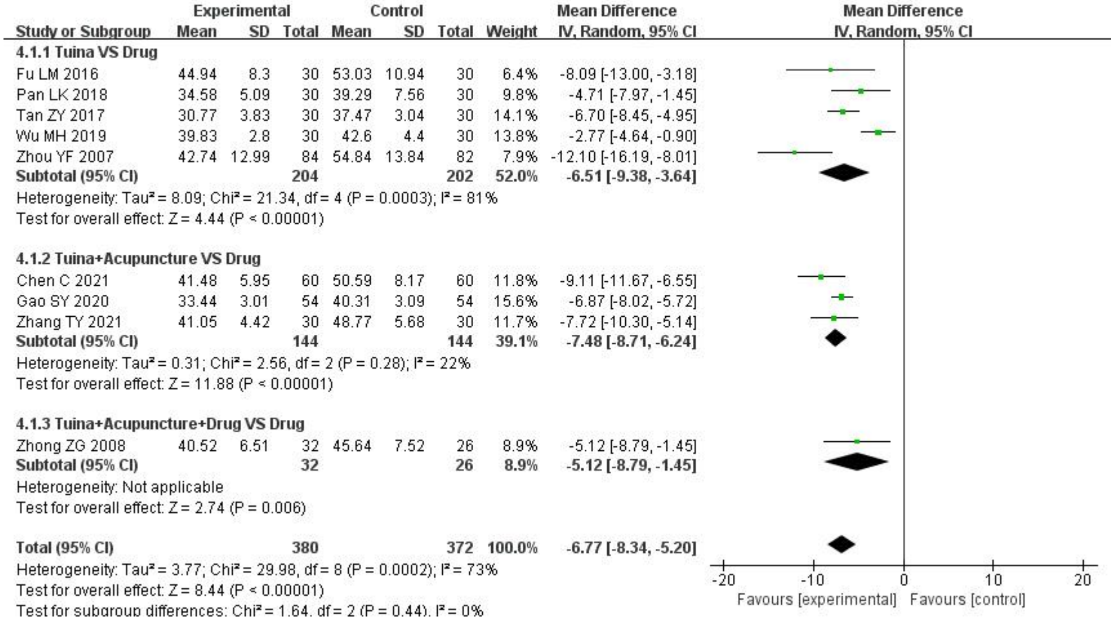


**Figure S8 Subgroup analysis of SAS**

| Study or Subgroup | Experimental Mean | Experimental SD | Experimental Total | Control Mean | Control SD | Control Total | Weight | Mean Difference IV, Random, 95% CI |
|---|---|---|---|---|---|---|---|---|
| Chen C 2021 | 32.16 | 4.42 | 60 | 34.51 | 7.03 | 60 | 17.4% | -2.35 [-4.45, -0.25] |
| Fu LM 2016 | 47.21 | 8.33 | 30 | 53.04 | 7.54 | 30 | 12.3% | -5.83 [-9.85, -1.81] |
| Gao SY 2020 | 34.56 | 2.38 | 54 | 41.23 | 2.47 | 54 | 19.9% | -6.67 [-7.58, -5.76] |
| Pan LK 2018 | 38.58 | 6.57 | 30 | 44.21 | 6.1 | 30 | 14.4% | -5.63 [-8.84, -2.42] |
| Wang YX 2016 | 43.36 | 11.17 | 87 | 55.96 | 12.8 | 89 | 13.5% | -12.60 [-16.15, -9.05] |
| Zhang TY 2021 | 44.74 | 6.68 | 30 | 53.26 | 7.45 | 30 | 13.5% | -8.52 [-12.10, -4.94] |
| Zhong ZG 2008 | 41.01 | 10.43 | 32 | 46.34 | 11.25 | 26 | 8.9% | -5.33 [-10.97, 0.31] |
| **Total (95% CI)** | | | **323** | | | **319** | **100.0%** | **-6.60 [-8.82, -4.37]** |

Heterogeneity: Tau² = 6.27; Chi² = 28.11, df = 6 (P < 0.0001); I² = 79%

Test for overall effect: Z = 5.80 (P < 0.00001)

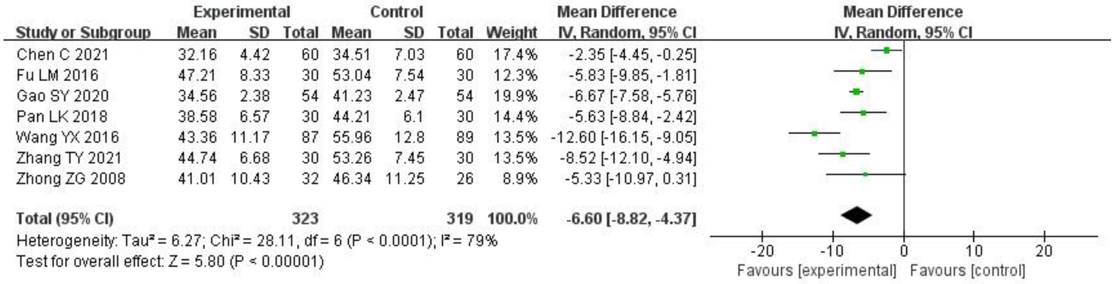


**Figure S9 Meta-analysis of SDS**

| Study or Subgroup | Experimental Mean | Experimental SD | Experimental Total | Control Mean | Control SD | Control Total | Weight | Mean Difference IV, Random, 95% CI |
|---|---|---|---|---|---|---|---|---|
| **5.1.1 Tuina VS Drug** | | | | | | | | |
| Fu LM 2016 | 47.21 | 8.33 | 30 | 53.04 | 7.54 | 30 | 12.3% | -5.83 [-9.85, -1.81] |
| Pan LK 2018 | 38.58 | 6.57 | 30 | 44.21 | 6.1 | 30 | 14.4% | -5.63 [-8.84, -2.42] |
| Wang YX 2016 | 43.36 | 11.17 | 87 | 55.96 | 12.8 | 89 | 13.5% | -12.60 [-16.15, -9.05] |
| **Subtotal (95% CI)** | | | **147** | | | **149** | **40.3%** | **-8.03 [-12.58, -3.48]** |
| Heterogeneity: Tau² = 12.78; Chi² = 9.68, df = 2 (P = 0.008); I² = 79% | | | | | | | | |
| Test for overall effect: Z = 3.46 (P = 0.0005) | | | | | | | | |
| **5.1.2 Tuina+Acupuncture VS Drug** | | | | | | | | |
| Chen C 2021 | 32.16 | 4.42 | 60 | 34.51 | 7.03 | 60 | 17.4% | -2.35 [-4.45, -0.25] |
| Gao SY 2020 | 34.56 | 2.38 | 54 | 41.23 | 2.47 | 54 | 19.9% | -6.67 [-7.58, -5.76] |
| Zhang TY 2021 | 44.74 | 6.68 | 30 | 53.26 | 7.45 | 30 | 13.5% | -8.52 [-12.10, -4.94] |
| **Subtotal (95% CI)** | | | **144** | | | **144** | **50.8%** | **-5.69 [-8.94, -2.43]** |
| Heterogeneity: Tau² = 6.90; Chi² = 15.48, df = 2 (P = 0.0004); I² = 87% | | | | | | | | |
| Test for overall effect: Z = 3.42 (P = 0.0006) | | | | | | | | |
| **5.1.3 Tuina+Acupuncture+Drug VS Drug** | | | | | | | | |
| Zhong ZG 2008 | 41.01 | 10.43 | 32 | 46.34 | 11.25 | 26 | 8.9% | -5.33 [-10.97, 0.31] |
| **Subtotal (95% CI)** | | | **32** | | | **26** | **8.9%** | **-5.33 [-10.97, 0.31]** |
| Heterogeneity: Not applicable | | | | | | | | |
| Test for overall effect: Z = 1.85 (P = 0.06) | | | | | | | | |
| **Total (95% CI)** | | | **323** | | | **319** | **100.0%** | **-6.60 [-8.82, -4.37]** |

Heterogeneity: Tau² = 6.27; Chi² = 28.11, df = 6 (P < 0.0001); I² = 79%

Test for overall effect: Z = 5.80 (P < 0.00001)

Test for subgroup differences: Chi² = 0.81, df = 2 (P = 0.67), I² = 0%

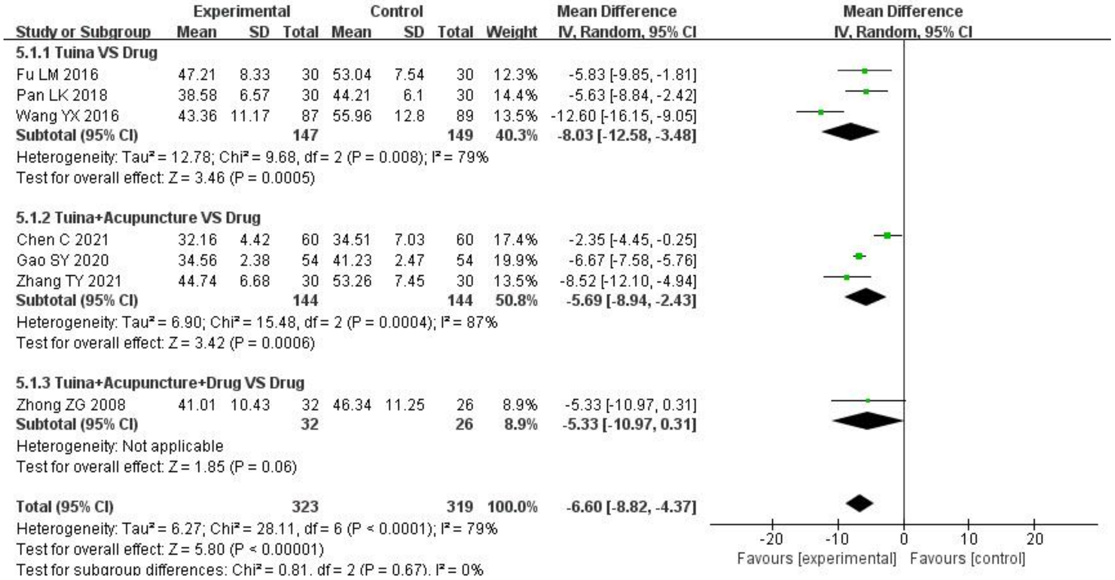


**Figure S10 Subgroup analysis of SDS**

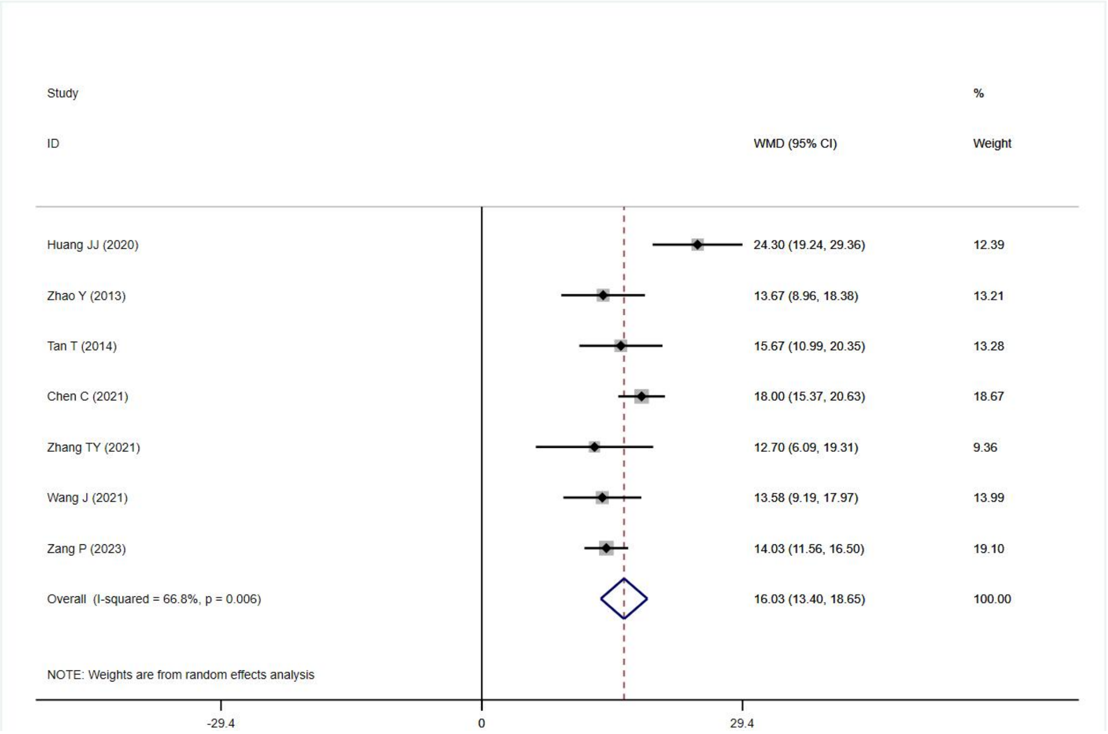


**Figure S11 Meta-analysis of 5-HT**

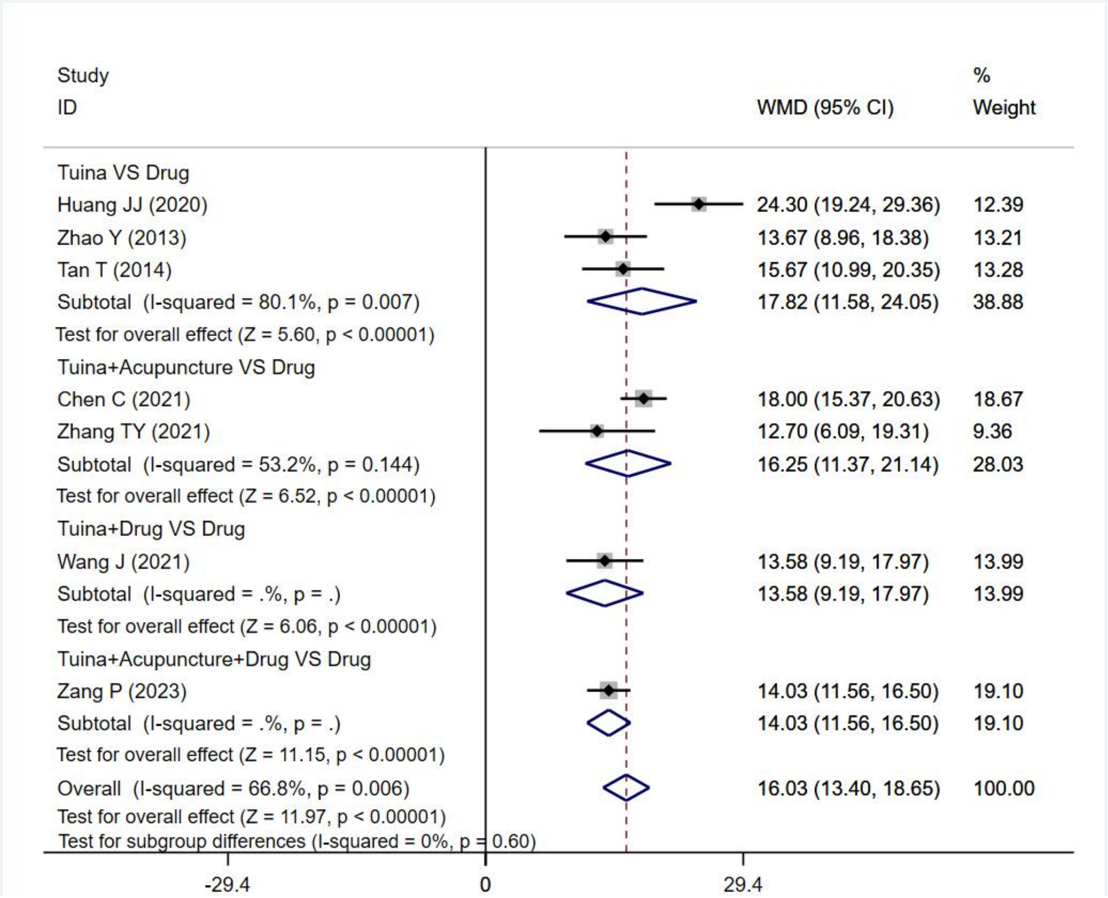


**Figure S12 Subgroup analysis of 5-HT**

**Results of sensitivity analysis**

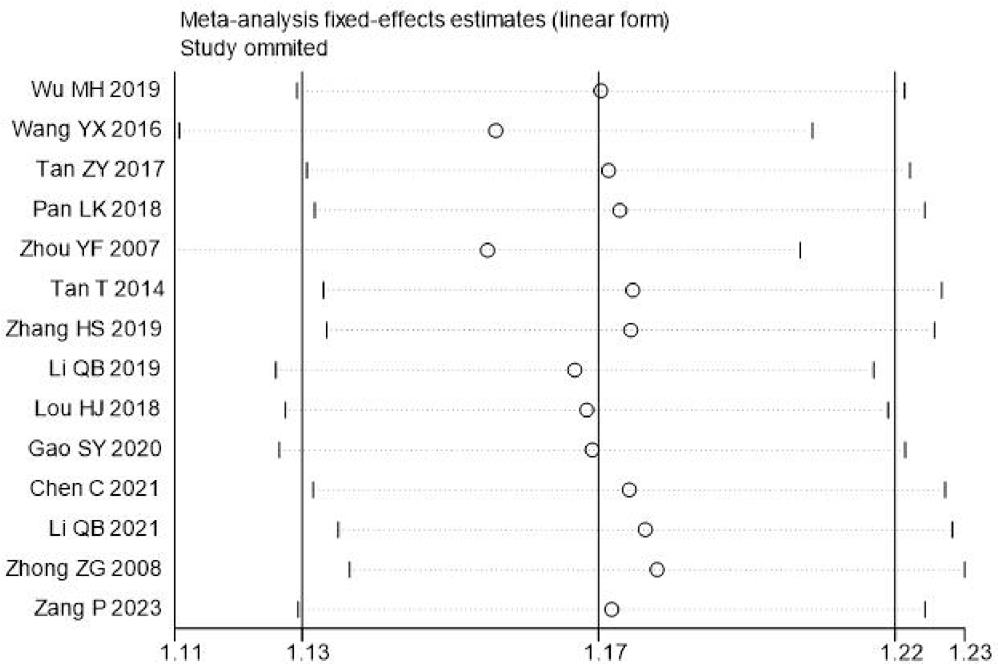


**Figure S13 Sensitivity analysis of total effective rate**

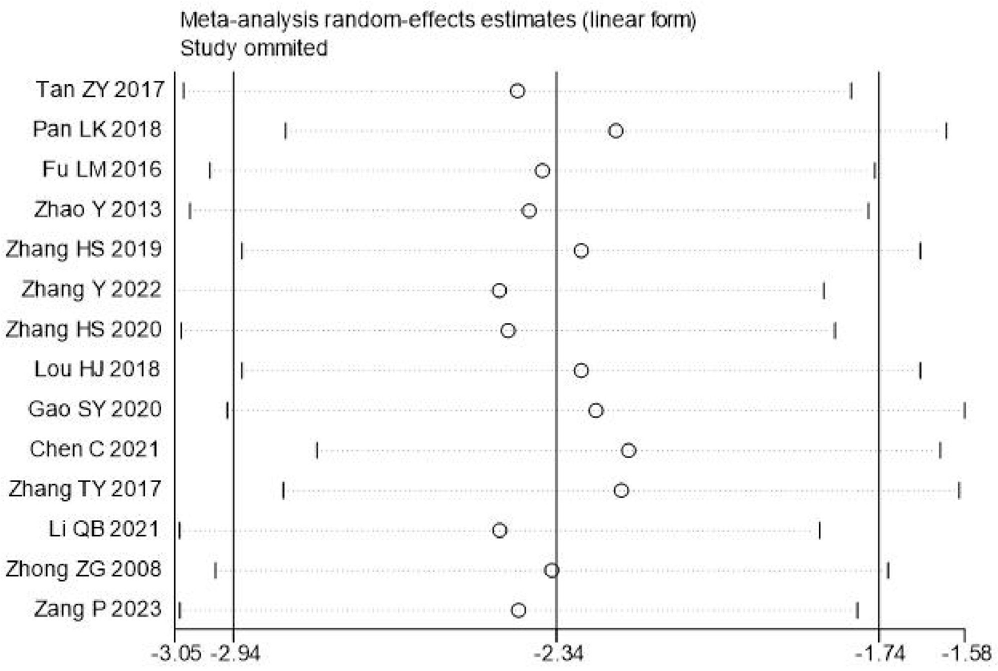


**Figure S14 Sensitivity analysis of PSQI**

**Table S1 Begger's test and Egger's test**

| Outcomes | Begger's test | | Egger's test | |
|---|---|---|---|---|
| | Z | P | t | P |
| Total effective rate | 0.05 | 0.956 | -0.32 | 0.758 |
| PSQI | -0.55 | 0.584 | -0.88 | 0.395 |
